# Design, Synthesis and Evaluation of Antioxidant and NSAID Derivatives with Antioxidant, Anti-Inflammatory and Plasma Lipid Lowering Effects

**DOI:** 10.3390/molecules29051016

**Published:** 2024-02-26

**Authors:** Panagiotis Theodosis-Nobelos, Gabriel Marc, Eleni A. Rekka

**Affiliations:** 1 Department of Pharmacy, School of Health Sciences, Frederick University, Nicosia 1036, Cyprus; 2 Department of Pharmaceutical Chemistry, Faculty of Pharmacy, “Iuliu Hațieganu” University of Medicine and Pharmacy, 41 Victor Babeș Street, RO-400010 Cluj-Napoca, Romania; marc.gabriel@umfcluj.ro; 3 Department of Pharmaceutical Chemistry, School of Pharmacy, Aristotelian University of Thessaloniki, 54124 Thessaloniki, Greece; rekka@pharm.auth.gr

**Keywords:** antioxidants, NSAID, anti-inflammatory, dyslipidemia, antioxidant activity, pluripotent derivatives

## Abstract

Amides containing methyl esters of γ-aminobutyric acid (GABA), L-proline and L-tyrosine, and esters containing 3-(pyridin-3-yl)propan-1-ol were synthesized by conjugation with 3,5-di-*tert*-butyl-4-hydroxybenzoic, an NSAID (tolfenamic acid), or 3-phenylacrylic (cinnamic, (*E*)-3-(3,4-dimethoxyphenyl)acrylic and caffeic) acids. The rationale for the conjugation of such moieties was based on the design of structures with two or more molecular characteristics. The novel compounds were tested for their antioxidant, anti-inflammatory and hypolipidemic properties. Several compounds were potent antioxidants, comparable to the well-known antioxidant, Trolox. In addition, the radical scavenging activity of compound **6** reached levels that were slightly better than that of Trolox. All the tested compounds demonstrated remarkable activity in the reduction in carrageenan-induced rat paw edema, up to 59% (compound **2**, a dual antioxidant and anti-inflammatory molecule, with almost 2.5-times higher activity in this experiment than the parent NSAID). Additionally, the compounds caused a significant decrease in the plasma lipidemic indices in Triton-induced hyperlipidemic rats. Compound **2** decreased total cholesterol by 75.1% and compound **3** decreased triglycerides by 79.3% at 150 μmol/kg (i.p.). The hypocholesterolemic effect of the compounds was comparable to that of simvastatin, a well-known hypocholesterolemic drug. Additionally, all compounds lowered blood triglycerides. The synthesized compounds with multiple activities, as designed, may be useful as potential candidates for conditions involving inflammation, lipidemic deregulation and oxygen toxicity.

## 1. Introduction

Systemic and local inflammation seem to be interrelated with many conditions, including atherosclerosis, a low-grade inflammatory disease, and neuroinflammation [1,2]. In all aspects of atherosclerosis, a complex network of implication of the innate and adaptive immune systems is involved, with macrophages, leukocytes, dendritic cells, and T- and B-lymphocytes modulating pro-inflammatory and anti-inflammatory cytokines, in favor of the former [3]. Such inflammatory and stressful cardiovascular risk factors generate a reduction in molecules with endothelial-regulating and neuroprotective properties, like NO, and an increase in the permeability of macromolecules, like cholesterol-containing lipoproteins, and more specifically, low-density-lipoprotein cholesterol (LDL-C), leading to additional triggering of inflammatory responses [4,5]. This lipid accumulation, under the parallel cytokine release, may result in necrosis and the formation of a lipid-rich necrotic core, separated from the arterial lumen, prolonging the release of inflammatory mediators from the periphery [6]. In this interplay, oxidative stress (OS) plays a key role, since pro-inflammatory cytokines bear indirect pro-oxidative potential, generating reactive species that deteriorate the functionality of the vascular wall. OS aggravates the inflammatory responses directly, and by the propagation of lipid and protein peroxidation. It also induces the production of oxidized LDL and formation of isoprostanes, prostaglandins and arachidonic acid, i.e., radical-induced peroxidation molecules, that further promote these conditions, leading to a vicious circle between OS, inflammation and lipid deregulation [7,8].

These peripheral inflammatory conditions may deteriorate the Blood Brain Barrier (BBB) integrity and lead to Central Nervous System (CNS) inflammatory responses, amplified by the activation of mast and glia cells. This is followed by increased Ca^++^ influx, BBB breakdown, cytokine release and secretion of corticotropin-releasing hormone (CRH) [9,10]. These inflammatory and oxidant effects may further deteriorate under the excitotoxic effects of glutamate and be reversed after the administration of antioxidant compounds [11]. The inflammatory and radical formation effects seem to be avoided by gamma aminobutyric acid (GABA, primary inhibitory neurotransmitter) and the activation of its receptors GABA_A_ and GABA_B_. Thus, T-cell proliferation is inhibited, Type-1 T helper (Th1) cell hypersensitivity responses are limited, and calcium cellular entry and T regulatory cells are promoted in a wide array of CNS and peripheral inflammatory diseases, like multiple sclerosis (MS), type 1 diabetes (T1D), rheumatoid arthritis, Sjogren’s syndrome, as well as inflammation in type 2 diabetes [12,13]. The brain injury deteriorates by hyperlipidemia with increases in IL-1, TNF-α and oxidized LDL in the brain and the serum, and with decreases in the levels of GABA and the relevant ratio of glutamate and GABA [14], damaging blood vessels and neurons [15]. In these oxidative conditions, pyridine moieties, like pyridine nucleotides and nicotinamide, result in reductive reactions, OS counteraction and DNA fragmentation inhibition [16]. Additionally, L-proline, another amino acid, seems to offer anti-inflammatory effects in the brain cortex and cerebellum in lipopolysaccharide-treated Wistar rats, with a decrease in oxidative stress parameters [17]. This confirms previous results on radical scavenging, and the anti-apoptotic and energy manipulation effects of L-proline [18,19].

Non-steroidal anti-inflammatory drugs (NSAIDs), through cyclooxygenase (COX) 1 and 2 inhibitory effects, possess anti-inflammatory, OS regulatory and hypolipidemic potencies that may address the atheromatic lesions in a positive multi-active manner [20,21]. However, the indirect, leukotriene-pathway-inducing effects may partly account for their adverse effects, and lipoxygenase inhibition may assist with this [22,23]. Cinnamic acid, on the other hand, and its methoxy and phenyl derivatives, like ferulic acid and caffeic acid, have been shown to offer cellular protective and antioxidant effects, with inflammation- and proliferation-reducing effects [24,25,26,27].

**Figure 1:**
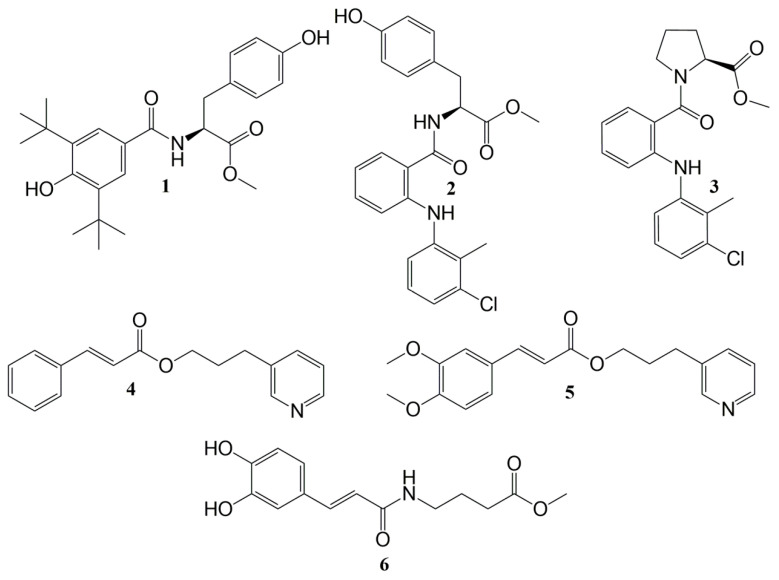
Structures of the synthesized compounds **1**–**6**.

**Figure 2:**
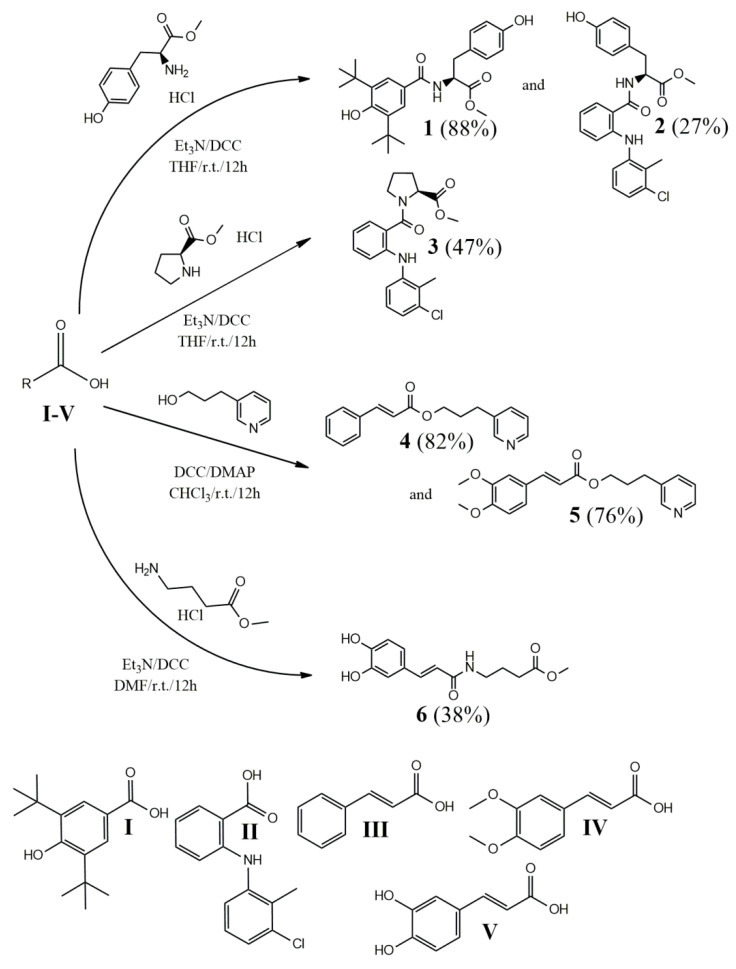
Synthesis of compounds **1**–**6**. THF: tetrahydrofuran; DMF: dimethylformamide; DCC: *N*,*N*′-dicyclohexylcarbodiimide; r.t.: room temperature; (): reaction yield.

In view of all the above, in this study, we synthesized a series of derivatives of L-tyrosine (compounds **1**, **2**), L-proline (compound **3**), 3-(pyridin-3-yl)propan-1-ol (compounds **4** and **5**) and GABA (compound **6**) (Figure 1). These compounds were amidated or esterified with 3,5-di-*tert*-butyl-4-hydroxybenzoic acid (compound I) (Figure 2), which is a molecule structurally related to butylated hydroxytoluene (BHT), a broadly used antioxidant with multiple activities and a low toxicity profile [28]. Further, tolfenamic acid (compound **II**), a well-applied NSAID, cinnamic acid (compound **III**), (*E*)-3-(3,4-dimethoxyphenyl)acrylic acid (compound **IV**), a 3,4-dimethoxy derivative of cinnamic acid and caffeic acid (compound **V**) were used. Finally, 3-(pyridin-3-yl)propan-1-ol esters have been shown to offer cellular protective and neurotrophic properties with anti-inflammatory activity [29,30].

The antioxidant activity of the synthesized compounds, expressed as inhibition of rat hepatic microsomal membrane lipid peroxidation, as well as interaction with the stable radical 2,2-diphenyl-1-picrylhydrazyl (DPPH), was evaluated. Their effects on carrageenan-induced rat paw edema, the in vitro inhibitory activity of soybean lipoxygenase and the hypolipidemic activity in vivo were also estimated. The aim of this study was to assess whether such hybrids, derived from parent compounds with specific characteristics like antioxidant or anti-inflammatory activities, could lead to multifunctional compounds with effects on OS and inflammation eradication, as well as lipid regulatory activity.

## 2. Results and Discussion

### 2.1. Synthesis

All the compounds were synthesized by direct amidation or esterification of the carboxylic group of the respective acids (compounds **I**–**IV**, Figure 2) with the methyl esters of L-tyrosine, L-proline and gamma-aminobutyric acid (GABA) or the 3-(pyridin-3-yl)propan-1-ol in yields that varied between 27 and 88%. For the tolfenamic acid derivatives (compounds **2** and **3**), and especially for compound **2**, the reaction was slow and occurred with lower yields. This was perhaps due to the steric hindrance of the bulky anhydride of the phenyl-amino-benzoyl moiety that inhibited the nucleophile insertion of tyrosine and proline. Cinnamic acid, and the (*E*)-3-phenylacrylic moiety in general, seemed to allow a facile insertion of the more flexible 3-pyridine propanol group, explaining the very good yields of compounds **4** and **5**. The use of the acyl-chloride or the anhydride (via DCC) were not shown to affect, in a different manner, the evolution of the esterification. 

### 2.2. Biological Evaluation

#### 2.2.1. Effect on Lipid Peroxidation

The effect of synthesized compounds **1**–**6** on rat hepatic microsomal membrane lipid peroxidation, expressed as IC_50_ values after 45 min of incubation, is shown in Table 1. Trolox was used as a reference compound for comparison.

Compounds **1** and **2** were active in this experiment and this may be partly due to the phenol group of tyrosine, although the phenyl ring of tyrosine is not accompanied by electron-donating groups, or highly extended conjugation, as is the case with the caffeic acid derivative (compound **6**) that bears the 3-phenylacrylate moiety and catechol structure. However, compound **6** did not offer a substantially differentiated decrease in lipid peroxidation, in comparison with compound **1**, since its increased polar area and its low lipophilicity may not facilitate the approach to the lipid phase (Table 2). This may also be the reason for the considerable difference in the activity between Trolox and compound **6**, although mechanistically, both of them have the ability to offer two hydrogen atoms as antioxidants. Compound **1** was almost three-fold more active than compound **2**, and this effect can be deduced from the dual electron-donating parts of compound **1**, the tyrosine and the 4-hydroxybenzoyl moieties, with the di-*tert*-butyl groups acting as electron donors via their inductive effect. This dual functionality may explain the increased activity of compound **1** in comparison to the parent acid (compound **I**) that has IC_50_ 257 μM in the same experiment [31].

On the other hand, compounds **3**, **4** and **5** did not show any antioxidant effect in lipid peroxidation, although they possess electron-donating groups and have the characteristics for approaching the lipid phase of the liver microsomal fraction, since they do not possess an easily abstracted hydrogen atom.

**Figure 3:**
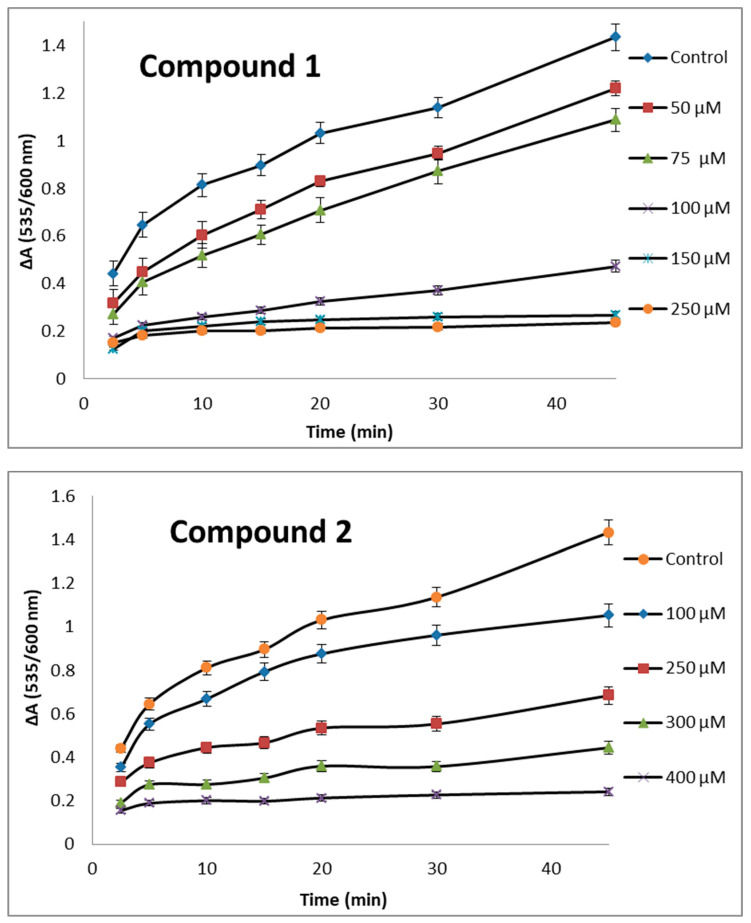

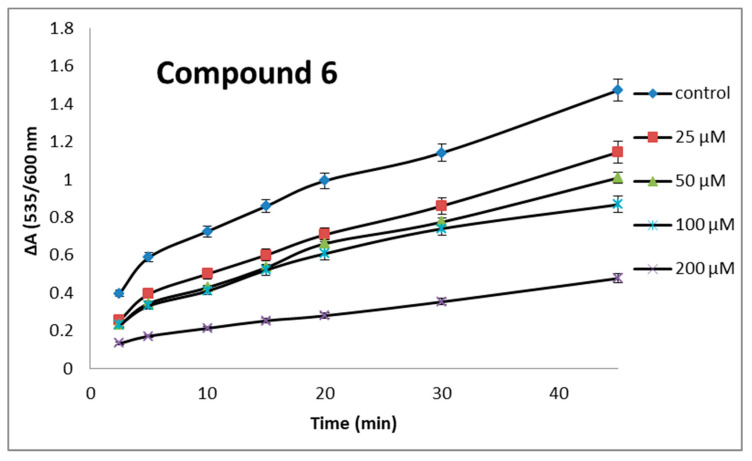
Effect of various concentrations of compounds **1**, **2** and **6** on lipid peroxidation.

The time course of lipid peroxidation, as affected by various concentrations of active compounds **1**, **2** and **6,** is shown in Figure 3.

#### 2.2.2. Interaction with DPPH

The antioxidant activity of compounds was also evaluated by their ability to interact with the stable, *N*-centered 1,1-diphenyl-2-picrylhydrazyl (DPPH) free radical. The percentage interaction between the compounds and DPPH, at various concentrations, is shown in Table 3.

Compounds **1**, **2** and **6** exhibited considerable activity against the DPPH radical, in agreement with the results from the lipid peroxidation inhibition assay. Compound **1** was approximately 2-fold more active than compound **2**. The reason for this may be the two phenol groups of compound **1**, when compared with compound **2**, which has only one phenol group. Compound **6** was the most active compound from the present series, which can be attributed to the catechol group. Also, compound **6** exhibited higher activity than Trolox, verifying its electron donating potential and its antioxidant effect that may partially be hindered by its polar characteristics. These results of caffeic acid derivative **6** are in accordance with previous studies [30]. However, the effect of 3,5-di-*tert*-butyl-4-hydroxybenzoic acid derivative **1**, in this assay, is improved compared to previously reported results [32,33]; although, in these studies the lipid peroxidation inhibition was increased. This accentuates the diversity of the effects of the clogP, TPSA and molecular volume on the approach and interaction with the lipids and the radicals in the reaction mixture of each experiment. Compounds **1**, **2** and **6** exhibited good radical scavenging ability, even in 8:1 proportions with DPPH. Also, compound **6** could fully neutralize the radical even at half the concentration of DPPH, ascertaining the dual hydrogen atom donating ability of the caffeic acid moiety and the relevant radical stabilization activity of the resulting phenyl radical. As was expected from the results of the lipid peroxidation assay, compounds **3**, **4** and **5** had no or very weak activity in this experiment, which can be correlated with their structure.

#### 2.2.3. Effect on Acute Inflammation

Carrageenan-induced paw edema is a well-known experimental protocol for acute inflammation, where, in the early phase, histamine, serotonin and kinin release take place, and in a later phase, neutrophil infiltration, and prostaglandin and cytokine release are involved [34]. 

The effect of the synthesized compounds on acute inflammation, applying the carrageenan rat paw edema model, as well as the anti-inflammatory activity of ibuprofen and tolfenamic acid, used as the reference, are shown in Table 4.

The compounds could reduce paw edema from 25% up to 59% at 150 μmol/kg i.p., 3.5 h after carrageenan administration. The most active compound, compound **2,** also had antioxidant capacity, in addition to the NSAID chemical moiety, indicating a possible implication of the antioxidant potential of the examined compounds in acute inflammation. Furthermore, the implication of the antioxidant effect of the compounds in this experiment may explain the fact that the following two most active anti-inflammatory compounds were compounds **1** and **6**, which are the derivatives with the best antioxidant activity among the synthesized compounds. The 3,5-di-*tert*-butyl-4-hydroxybenzoic and caffeic acid derivatives have also been shown previously to possess good, and in cases excellent, anti-inflammatory activity, and this seems to be partly dependent on their effects against oxidative stressful conditions and on inflammation-related enzymes [30,35].

Tolfenamic acid and NSAIDs in general, may, in some cases, adversely affect the production of reactive oxygen species, leading to apoptosis and adverse effects [36]. This may be reversed by the antioxidant potential of the tyrosine moiety and offer a high synergistic effect, as far as the activity of compound **2** shows, but also diminishing the unwanted effects of the parent NSAID [36,37]. Fenamates have also been shown to inhibit calcium influx in isolated mitochondria and polymorphonuclear leukocytes [38], offering improvement in various disorders characterized by pain and inflammation [39,40]. Furthermore, when co-administered with other anti-inflammatory and cellular protective molecules, it has been proven to offer synergistic anti-inflammatory effects [41]. This may be the case with both compounds **2** and **3** that have almost a 2.5- and 1.5-fold increased anti-inflammatory activity, respectively, compared with the parent acid. 

As for compounds **4** and **5**, their activity was similar to and comparable with the well-established NSAIDs, ibuprofen and tolfenamic acid. Cinnamic acid and an array of its derivatives have been found to decrease carrageenan-induced inflammation, suppressing factors such as iNOS, COX-2 and NF-κB expression, as well as TNF-α [42,43]. Furthermore, we have shown that these 3-phenylacrylate moieties seem to enhance the anti-inflammatory potency in vivo and offer more than one pharmacological activity [32,33,35], whilst 3-(pyridin-3-yl)propan-1-ol insertion may offer movement towards this direction [30,44]. 

#### 2.2.4. Effect on Lipoxygenase

Eicosanoids form an integral part of homeostasis of the organism and are synthesized from arachidonic acid, mainly via two pathways. The former implicates COX enzymes and the latter, the production of leukotrienes by lipoxygenases (LOX). Both these processes are also involved in various inflammatory reactions, like rheumatoid arthritis, and cardiovascular and neurodegenerative events [45,46]. Additionally, LOX inhibition may assist with decreasing cellular lipid accumulation and foam cell formation [47]. The inhibition of soybean lipoxygenase by the synthesized compounds is shown in Table 5. Tolfenamic acid and nordihydroguaiaretic acid (NDGA) were used as reference compounds for comparison. 

All the compounds had medium or low activity in this experiment. Compound **2,** with good antioxidant, radical scavenging and in vivo anti-inflammatory potential, showed the best lipoxygenase inhibition activity. This was followed by compound **5**, which, despite the absence of antioxidant activity, had a moderate anti-inflammatory effect in vivo, an effect that may partially be related to the lipoxygenase inhibitory activity of the compound. Compound **3** was found to be inactive, while for compound **4**, some modest activity was identified, although this was deprived of any direct antioxidant effect, as was the case with our previous findings concerning cinnamic derivatives [32,33]. 

Lastly, it can be deduced that our compounds act in a competitive manner with linoleic acid, since no inhibition could be detected using linoleic acid at a 1 mM concentration (higher than the saturating substrate concentration) under the same experimental setting. Additionally, the compounds may not affect the free radicals formed by LOX, but instead affect the binding of the substrate at the active center of the enzyme, since their antioxidant effect does not closely relate with their LOX inhibitory effect. This may partially explain the low activity of compounds **1** and **6**, despite their good antioxidant effect. A possible reason for this low inhibitory activity may be the bulky structure of the di-*tert*-butylphenyl moiety of compound **1** and the polarity of both compounds that do not induce an effective approach to the U-shaped lipophilic channel of the enzyme.

#### 2.2.5. Effect of the Synthesized Compounds on Hyperlipidemia

Lipid levels are interrelated with systemic inflammation, and the differentiation of one may result in changes in the other, increasing the risk of cardiovascular events and plaque formation [48]. Thus, the lipid-lowering and vascular protective effects of some compounds have been shown to also be related to the reduction in pro-inflammatory cytokines and chemokines, and the inactivation of macrophage infiltration, at the vascular and tissue levels [49,50,51].

The synthesized compounds were tested for their anti-dyslipidemic activity in Triton-induced hyperlipidemia to rats. The systemic administration of Triton-WR1339 to rats results in the elevation of plasma cholesterol, intermediate density lipoproteins, LDL cholesterol and, especially, triglyceride levels. Results of the effect of our compounds, together with simvastatin and tolfenamic acid, on Triton WR1339 (tyloxapol) induced hyperlipidemia are shown in Table 6.

All the tested compounds caused statistically significant decreases in total cholesterol and triglycerides, reaching up to 75.1% and 79.3% for each marker, respectively. With the exception of compound **2**, all the compounds reduced both lipidemic indices in a similar manner.

The good activity of compounds **1**, **2** and **6** may partly rely on their antioxidant and in vivo anti-inflammatory activities, and this is perhaps based on their whole molecular entity, since tolfenamic acid alone has much lower hypolipidemic activity [21]. Compound **2** was comparable to simvastatin, a well-known HMG-CoA reductase inhibitor, in decreasing cholesterol. In addition, it produced a very good improvement in triglycerides, while the effect of the statin on triglycerides was negligible. Thus, the multi-functionality of these compounds may assist with the improved management of lipids, allowing the organism to cope with them, in a state of a decreased inflammation and oxidative insult. Ιnterestingly, compound **3** was the most active, highly decreasing both indices. Although it does not bear antioxidant or increased anti-inflammatory characteristics, compound **3** was very active in this experiment. The participation of the proline structure may be involved, since it has been shown to offer hypolipidemic potency in vivo [52]. Similarly, compounds **4** and **5** also considerably lowered lipid levels, although they presented weak or no activity in all other experiments. It could be suggested that the (methoxy)cinnamyl moiety is responsible for this effect, since it has been reported that many cinnamic acid derivatives possessed hypolipidemic activity [53,54]. Of course, other factors may be related to this lipid decrease in vivo.

### 2.3. In Silico Calculations

Density Functional Theory (DFT) has changed the landscape of material science, particularly the field of antioxidants design, offering a robust theoretical framework to comprehend molecules at the atomic level through electronic property calculations. This renders it indispensable for predicting antioxidant behavior. Through simulations that analyze the electronic structure and energy of molecules, along with potential resultant radicals, DFT proves instrumental in identifying potent antioxidants with effective free-radical scavenging capabilities. In this section, the data resulting from the in silico DFT calculation will be presented to clarify how the structure of the compounds and the position of the OH groups influence the antioxidant reactivity of compounds **1**–**6**.

The choice to perform the evaluations in more environments was made because it can provide valuable insights into the behavior and effectiveness of the compounds under different conditions. Interaction of the studied molecules with the molecules of the solvent having different chemical and electronic properties can strongly influence the behavior of compounds.

For the studied compounds, the computed HOMO and LUMO frontier orbitals in vacuum, non-polar solvent and water are presented in Table 7. The frontier orbitals are involved in electron transfer. HOMO represents the highest energy level where electrons are located and LUMO represents the lowest energy level with unoccupied electron states. It is desirable for an antioxidant to have low HOMO levels and high LUMO levels to be effective in neutralizing free radicals.

Overall, for the studied compounds, water tends to shift both frontier orbital levels to lower states, while in a non-polar environment, they tend to increase. Combining the two effects, the effect on the HOMO–LUMO gap in a non-polar environment is increasing (reducing the reactivity of the compounds), while water tends to decrease it (increasing the reactivity of the compounds).

If we divide the compounds into active (**1**, **2** and **6**) and inactive (**3**, **4** and **5**) groups according to the results of the in vitro antioxidant assays, it would be obvious from a simple analysis that the energy levels of the frontier molecular orbitals and the gap between them cannot be correlated with the results from the antioxidant assays. 

Visual analysis of the position of the HOMO and LUMO orbitals (Table 8) would shed light on why those two parameters cannot accurately describe the antioxidant properties of the compounds. 

One suggestive observation regarding this aspect can be made for compound **1**, where HOMO is situated over the tyrosine fragment, while LUMO is situated over the di-*tert*-butylphenol fragment. But, the best exemplification that can be made is for compound **2**, where both HOMO and LUMO are found over the *N*,*N*-diphenylamino region of the molecule and not over the tyrosine fragment, which is the main antioxidant fragment of the molecule. 

For compounds **4**, **5** and **6**, which share a high similarity over the phenyl-propenone fragment and where both HOMO and LUMO are found, the high antioxidant activity for compound **6** in the in vitro assays is correlated with the lowest HOMO between them in all three environments. 

Since, as stated above, the evaluation of the frontier orbitals HOMO and LUMO cannot always describe the reactivity of the antioxidants, evaluation of the bond dissociation enthalpy (BDE) was performed, as a very sensitive descriptor of the antioxidant activity for the compounds that can release hydrogen atoms (**1**, **2** and **6**) (Table 9). For compounds **3**, **4** and **5,** it was considered that they cannot release hydrogen atoms under normal conditions due to all hydrogen-atom bondings being strong (C-H or N-H), making it difficult for them to be extracted as antioxidants under physiological conditions.

Performing a classification of the results from the computed BDE for the OH groups of compounds **1**, **2** and **6,** it can be identified that compound **6** has the lowest values from the current series, for both OH groups. In compound **1,** the di-*tert*-butylphenol OH group exhibited an intermediary BDE in the current series, while the tyrosine OH group exhibited the lowest BDE in both compounds **1** and **2**. The order of BDE for OH groups in the three compounds was identical, independent of the environment tested. This order of OH BDE is very highly correlated with the antioxidant activity.

When analyzing how the solvent affects the reactivity of the OH groups, the tyrosine OH from compounds **1** and **2** is the least influenced by the environment, being favored by 0.95 kcal/mol in a non-polar environment. The di-*tert*-butylphenol group from compound **1** will break with 1.83 kcal/mol more, in water than in vacuum. Meanwhile, in a non-polar environment, it is even favored, breaking with 0.36 kcal/mol less, than in vacuum. 

For compound **6**, the *para* OH group is more reactive than the *meta* one, with a difference of 2.61 kcal/mol in vacuum, 2.80 kcal/mol in a non-polar environment and 3.23 kcal/mol in water. The strongest negative effect of water against hydrogen atom release was identified for the catechol groups of compound **6**. As expected, due to the properties of the phenoxy radical, in the vacuum and a non-polar environment, the reaction would occur more easily than in water with 2.75 kcal/mol for the *para* OH and 3.36 kcal/mol for the *meta* OH.

## 3. Materials and Methods

All commercially available chemicals of the appropriate purity were purchased from Merck (Kenilworth, NJ, USA) or Sigma (St. Louis, MO, USA). The IR spectra were recorded on a Perkin Elmer Spectrum BX FT-IR spectrometer (Waltham, MA, USA). The ^1^H NMR and ^13^C NMR spectra were recorded using an AGILENT DD2-500 MHz (Santa Clara, CA, USA) spectrometer. All chemical shifts are reported in δ (ppm) and signals are given as follows: s, singlet; d, doublet; t, triplet; m, multiplet. Melting points (m.p.) were determined with a MEL-TEMPII (Laboratory Devices, Sigma-Aldrich, Milwaukee, WI, USA) apparatus. The microanalyses were performed on a Perkin-Elmer 2400 CHN elemental analyzer (Waltham, MA, USA). Thin-layer chromatography (TLC silica gel 60 F254 aluminum sheets, Merck, Kenilworth, NJ, USA) was used to monitor the evolution of the reactions and the spots were visualized under UV light.

κ-Carrageenan and lipoxygenase type I-B from soybean were purchased from Sigma (St. Louis, MO, USA). For the in vivo experiments, Wistar rats (160–220 g, 3–4 months old) were kept in the Centre of the School of Veterinary Medicine (EL54 BIO42), Aristotelian University of Thessaloniki, which is registered by the official state veterinary authorities (presidential degree 56/2013, in harmonization with the European Directive 2010/63/EEC). The experimental protocols were approved by the Animal Ethics Committee of the Prefecture of Central Macedonia (no. 270079/2500).

### 3.1. Synthesis

General method for the synthesis of compounds **1**–**3** and **6**

Acids (1 mmol) were dissolved in dry THF (tetrahydrofuran, 10 mL), or DMF (dimethylformamide, 3 mL, in case of caffeic acid). The corresponding methyl esters of amino acid hydrochloride (1.2 mmol) L-tyrosine, L-proline or gamma-aminobutyric acid were suspended in the same solvent and triethylamine (1.3 mmol) was added. The mixture was stirred for 10 min, *N*,*N*′-dicyclohexylcarbodiimide (DCC, 1.3 mmol) was added and the final mixture was left under stirring in a nitrogen atmosphere overnight, at room temperature. The resulting mixture was filtered, washed successively with water and 5% NaHCO_3_ solution and dried over Na_2_SO_4_, and the final compounds were isolated with flash chromatography, using petroleum ether and ethyl acetate as eluents.

Synthesis of compound **4**

In a solution of the corresponding cinnamyl chloride (1 mmol) in dry CH_2_Cl_2_ (10 mL), the 3-(pyridin-3-yl)propan-1-ol (1.1 mmol) and triethylamine (1.2 mmol) were added at 0 °C. The mixture was left at room temperature under a nitrogen atmosphere for 3 h, then washed successively with water and 5% aqueous NaHCO_3_ solution, and dried over Na_2_SO_4_. The final compound was isolated with flash chromatography.

Synthesis of compound **5**

In a solution of the corresponding acid (1 mmol) in dry CH_2_Cl_2_ (10 mL), 3-(pyridin-3-yl)propan-1-ol (1.2 mmol), 4-dimethylamino-pyridine (DMAP, 0.1 mmol) and *N*,*N*′-dicyclohexylcarbodiimide (DCC, 1.2 mmol) were added and the mixture was stirred under a nitrogen atmosphere overnight, at room temperature. The resulting mixture was filtered, washed successively with water and 5% NaHCO_3_ solution and dried over Na_2_SO_4_, and the final compound was isolated with flash chromatography, using petroleum ether and ethyl acetate as eluents.

*(S)-Methyl 2-(3,5-di-tert-butyl-4-hydroxybenzamido)-3-(4-hydroxyphenyl)propanoate* (compound **1**):

Flash chromatography (petroleum ether/ethyl acetate 2:1). White solid, yield 88%, m.p. 175–176 °C. IR (Nujol) λ_max_: 3579 (O-H), 3381 (N-H), 1731 (C=O ester), 1630 (C=O amide), 1599, 1576 (C-C aromatic) cm^−1^. ^1^H NMR (CDCl_3_ + DMSO-d6) δ (ppm): 1.30 (s, 18H, -CH_3_), 2.98 (d, 2H, *J* = 6.1 Hz, -NHCH-C**H_2_**-), 3.60 (s, 3H, -O-C**H_3_**), 4.75 (dd, 1H, *J* = 13.2, 6.1 Hz, -NHC**H**-CH_2_-), 6.63 (d, 1H, *J* = 8.3 Hz, aromatic tyrosine C3, C5 H), 6.72 (d, 1H, *J* = 7.5 Hz, -N**H**-), 6.87 (d, 2H, *J* = 8.3 Hz, aromatic tyrosine C2, C5 H), 7.41 (s, 2H, aromatic di-*tert*-phenol C2, C6 H). ^13^C-NMR (CDCl_3_) δ (ppm): 30.05 (C-(**C**H_3_)_3_), 34.35 (**C**-(CH_3_)_3_), 37.23 (NH-CH-**C**H_2_-Ar), 52.41 (COO**C**H_3_), 53.50 (NH-**C**H-CH_2_-Ar), 115.65 (tyrosine phenyl **C3**, **C5**), 124.34 (di-tert-butyl-phenyl **C2**, **C6**), 124.77 (tyrosine phenyl **C1**), 127.06 (tyrosine phenyl **C2**, **C6**), 130.32 (di-tert-butyl-phenyl **C1**), 135.93 (di-tert-butyl-phenyl **C3**, **C5**), 155.58 (di-*tert*-butyl-phenyl **C4**), 157.18 (tyrosine phenyl **C4**), 167.98 (**C**ONH), 172.49 (**C**OOCH_3_). Anal. Calcd for C_25_H_33_NO_5_: C, 70.23; H, 7.78; N, 3.28. Found: C, 69.89; H, 7.90; N, 3.11.

*(S)-Μethyl 2-(2-((3-chloro-2-methylphenyl)amino)benzamido)-3-(4-hydroxyphenyl)propanoate* (compound **2**):

Flash chromatography (petroleum ether/ethyl acetate 3:1 and subsequently 2:1). White solid, yield 27%, m.p. 134 °C. IR (Nujol) λ_max_: 3556 (O-H), 3344 (N-H), 1726 (C=O ester), 1629 (C=O amide), 1613, 1578 (C-C aromatic) cm^−1^. ^1^H NMR (CDCl_3_ + DMSO-d6) δ (ppm): 2.23 (s, 3H, phenyl-C**H_3_**), 3.06 (t, 2H, *J* = 6.1 Hz, -NHCH-C**H_2_**-), 3.66 (s, 3H, -O-C**H_3_**), 4.83 (dd, 1H, *J* = 13.1, 6.1 Hz, -NHC**H**-CH_2_-), 6.64 (d, 1H, *J* = 7.2 Hz, aromatic amino methyl phenyl C6 H), 6.68 (d, 2H, *J* = 8.4 Hz, aromatic tyrosine C3, C5 H), 6.83 (d, 1H, *J* = 8.5 Hz, aromatic amino methyl phenyl C4 H), 6.90 (d, 2H, *J* = 8.4 Hz, aromatic tyrosine C2, C6 H), 7.07–6.95 (m, 2H, aromatic benzoic acid C5 H and aromatic amino methyl phenyl C5 H), 7.18–7.07 (m, 2H, aromatic benzoic acid C4, C6 H), 7.32 (d, 1H, *J* = 7.8 Hz, aromatic amino methyl phenyl C3 H). ^13^C-NMR (CDCl_3_) δ (ppm): 24.87 (Ar-CH_3_), 33.86 (NH-CH-**C**H_2_-Ar), 52.51 (-O-CH_3_), 62.75 (NH-**C**H-CH_2_-Ar), 115.63 (tyrosine phenyl **C3**, **C5**), 117.84 (3-chloro-2-methylphenyl **C6**), 118.83 (benzoyl **C3**), 119.67 (3-chloro-2-methylphenyl **C4**), 120.65 (benzoyl **C1**), 124.44 (benzoyl **C5**), 125.38 (3-chloro-2-methylphenyl **C2**), 126.71 (benzoyl **C6**), 130.40 (tyrosine phenyl **C1**), 132.60 (tyrosine phenyl **C2**, **C6**), 135.43 (benzoyl **C4**), 141.12 (3-chloro-2-methylphenyl **C3**), 143.14 (benzoyl **C2**), 146.19 (3-chloro-2-methylphenyl **C1**), 155.32 (tyrosine phenyl **C4**), 168.87 (Ar**C**=O), 172.16 (**C**OOCH_3_). Anal. Calcd for C_24_H_23_ClN_2_O_4_: C, 65.68; H, 5.28; N, 6.38. Found: C, 65.56; H, 5.12; N, 6.30.

*(S)-Methyl 1-(2-((3-chloro-2-methylphenyl)amino)benzoyl)pyrrolidine-2-carboxylate* (compound **3**)

Flash chromatography (petroleum ether/ethyl acetate 5:1 and subsequently 3:1). Viscous transparent oil, yield 47%. IR λ_max_: 3344 (N-H), 3066 (aromatic C-H), 2980, 2952, 2879 (alkyl C-H), 1743 (C=O ester), 1625 (C=O amide), 1582 (C-C aromatic) cm^−1^. ^1^H NMR (CDCl_3_), δ (ppm): 2.20–1.86 (m, 4H, pyrrolidine C2,C3 H), 2.35 (s, 3H, -C**H_3_**), 3.64 (s, 2H, pyrrolidine C4 H), 3.77 (s, 3H, -O-C**H_3_**), 4.73 (s, 1H, pyrrolidine C1 H), 6.84 (t, 1H, *J* = 7.4 Hz, aromatic 3-chloro-2-methylphenyl C5 H), 7.00 (d, 1H, *J* = 8.3 Hz, aromatic 3-chloro-2-methylphenyl C6 H), 7.15–7.03 (m, 2H, aromatic benzoyl C5 H and aromatic 3-chloro-2-methylphenyl C4 H), 7.30–7.15 (m, 2H, aromatic benzoyl C4, C6 H), 7.35 (d, 1H, *J* = 11.8 Hz, aromatic benzoyl C3 H). ^13^C-NMR (CDCl_3_) *δ*: 14.71 (Ar-**C**H_3_), 25.31 (pyrrolidine **C4**), 29.33 (pyrrolidine **C3**), 49.84 (pyrrolidine **C5**), 52.41 (-O-**C**H_3_), 58.82 (pyrrolidine **C2**), 115.49 (3-chloro-2-methylphenyl **C6**), 115.53 (benzoyl **C3**), 118.51 (benzoyl **C5**), 119.39 (benzoyl **C1**), 122.64 (3-chloro-2-methylphenyl **C4**), 123.76 (3-chloro-2-methylphenyl **C5**), 126.66 (3-chloro-2-methylphenyl **C2**), 128.18 (benzoyl **C6**), 130.83 (benzoyl **C4**), 134.93 (3-chloro-2-methylphenyl **C3**), 135.43 (benzoyl **C2**), 141.64 (3-chloro-2-methylphenyl **C1**), 167.54 (Ar**C**=O), 172.80 (CH**C**=O). Anal. Calcd for C_20_H_21_ClN_2_O_3_ × 0.9H_2_O: C, 61.74; H, 5.91; N, 7.20. Found: C, 61.86; H, 5.93; N, 7.03.

*3-(Pyridin-3-yl)propyl cinnamate* (compound **4**):

Flash chromatography (petroleum ether/ethyl acetate 3:1). Viscous transparent oil, yield 82%. IR λ_max_: 3406 (N-H), 1711 (C=O ester), 1637 (C=C), 1576 (C-C aromatic) cm^−1^. ^1^H NMR (CDCl_3_), δ (ppm): 2.09–2.01 (m, 2H, -CH_2_-C**H_2_**-CH_2_-), 2.75 (t, 2H, *J* = 7.6 Hz, -CH_2_-CH_2_-C**H_2_**-), 4.24 (t, 2H, *J* = 6.4 Hz, -O-C**H_2_**-CH_2_-CH_2_-), 6.44 (d, 1H, *J* = 16.0 Hz, CH=C**H**-C=O), 7.22 (dd, 1H, J = 7.7, 4.8 Hz, C5 H pyridine), 7.41–7.36 (m, 3H, C3–C5 aromatic cinnamyl H), 7.55–7.51 (m, 3H, C4 H pyridine and C2, C3 aromatic cinnamyl H), 7.67 (d, 1H, *J* = 16.0 Hz, -C**H**-CH-C=O), 8.45 (dd, 1H, *J* = 4.8, 1.5 Hz, C6 H pyridine), 8.55 (d, 1H, *J* = 2.0 Hz, C2 H pyridine). ^13^C NMR (CDCl_3_) δ (ppm): 29.48 (1C, -CH_2_-**C**H_2_-CH_2_-), 29.99 (1C, -CH_2_-CH_2_-**C**H_2_-), 63.51 (1C, -**C**H_2_-CH_2_-CH_2_-), 117.85 (1C, CH=**C**H-C=O), 123.40 (1C, C5 pyridine), 128.08 (2C, **C2**, **C6** phenyl), 128.89 (2C, **C3**, **C5** phenyl), 130.34 (1C, **C4** phenyl), 134.30 (1C, **C1** phenyl), 135.92 (1C, **C4** pyridine), 136.53 (1C, **C3** pyridine), 144.95 (1C, **C**H=CH-C=O), 147.49 (1C, **C6** pyridine), 149.82 (1C, **C2** pyridine), 166.91 (1C, CH=CH-**C**=O). Anal. Calcd for C_17_H_17_NO_2_: C, 76.38; H, 6.41; N, 5.24. Found: C, 76.69; H, 6.80; N, 5.28.

*(E)-3-(Pyridin-3-yl)propyl 3-(3,4-dimethoxyphenyl)acrylate* (compound **5**):

Flash chromatography (petroleum ether/ethyl acetate 5:1 and subsequently 2:1). White solid, yield 76%. IR (Nujol) λ_max_: 3378 (N-H), 1699 (C=O ester), 1634 (C=C), 1598, 1582 (C-C aromatic) cm^−1^. ^1^H NMR (CDCl_3_), δ (ppm): 2.07–2.00 (m, 2H, -CH_2_-C**H_2_**-CH_2_-), 2.75 (t, 2H, *J* = 7.6 Hz, -CH_2_-CH_2_-C**H_2_**-), 3.91, 3.90 (s, 6H, -OCH_3_), 4.22 (t, 2H, *J* = 6.4 Hz, -O-C**H_2_**-CH_2_-CH_2_-), 6.30 (d, 1H, *J* = 15.9 Hz, CH=C**H**-C=O), 6.86 (d, 1H, *J* = 8.3 Hz, phenyl C5), 7.05 (d, 1H, *J* = 1.9 Hz, phenyl C2), 7.10 (dd, 1H, *J* = 8.3, 1.9 Hz, phenyl C6), 7.22 (dd, 1H, J = 7.7, 4.8 Hz, C5 H pyridine), 7.54–7.51 (m, 3H, C4 H pyridine), 7.60 (d, 1H, *J* = 15.9 Hz, -C**H**-CH-C=O), 8.45 (dd, 1H, *J* = 4.8, 1.4 Hz, C6 H pyridine), 8.49 (d, 1H, *J* = 1.8 Hz, C2 H pyridine). ^13^C NMR (CDCl_3_) δ (ppm): 29.48 (1C, -CH_2_-**C**H_2_-CH_2_-), 30.02 (1C, -CH_2_-CH_2_-**C**H_2_-), 55.96, 55.87 (2C, -O**C**H_3_), 63.37 (1C, -**C**H_2_-CH_2_-CH_2_-), 109.52 (1C, C2 phenyl), 110.97 (1C, C5 phenyl), 115.49 (1C, CH=**C**H-C=O), 122.67 (1C, **C6** phenyl), 123.39 (1C, **C5** pyridine), 127.26 (1C, **C1** phenyl), 135.91 (1C, **C4** pyridine), 136.56 (1C, C3 pyridine), 144.86 (1C, **C**H=CH-C=O), 147.55 (1C, **C6** pyridine), 149.83 (1C, **C2** pyridine), 149.16 (1C, **C3** phenyl), 151.13 (1C, **C4** phenyl), 167.14 (1C, CH=CH-**C**=O). Anal. Calcd for C_19_H_21_ΝO_4_: C, 69.71; H, 6.47; Ν, 4.28. Found: C, 69.69; H, 6.74; Ν, 4.44.

*(E)-Methyl 4-(3-(3,4-dihydroxyphenyl)acrylamido)butanoate* (compound **6**):

Flash chromatography (petroleum ether/ethyl acetate 1:1 and subsequently ethyl acetate alone). Semisolid, yield 38%. IR λ_max_: 3498, 3366 (O-H), 3327 (N-H), 1734 (C=O ester), 1656 (C=O amide), 1627 (C=C), 1598, 1577, 1546 (C-C aromatic) cm^−1^. ^1^H NMR (CDCl_3_ + DMSO-d6) δ (ppm): 2.25 (t, 2H, *J* = 7.4 Hz, NH-CH_2_-CH_2_-C**H_2_**-C=O), 3.25–3.19 (m, 2H, NH-CH_2_-C**H_2_**-CH_2_-C=O), 3.40–3.33 (m, 2H, NH-C**H_2_**-CH_2_- CH_2_-C=O), 3.52 (s, 3H, -OC**H_3_**), 5.19 (s, 2H, -**OH**), 6.14 (d, 1H, *J* = 15.6 Hz, -C**H**=CH-C0), 6.68 (d, 1H, *J* = 8.2 Hz, aromatic di-hydroxy-phenyl C5 H), 6.74 (dd, 1H, *J* = 8.2, 2.2 Hz, aromatic di-hydroxy-phenyl C6 H), 6.74 (s, 1H, NH) 6.90 (d, 1H, *J* = 2.2 Hz, aromatic di-hydroxy-phenyl C2 H), 7.29 (d, 1H, *J* = 15.6 Hz, -CH=C**H**-CO). ^13^C NMR (CDCl_3_ + DMSO-d6) *δ*: 29.54 (1C, NH-CH_2_-**C**H_2_-CH_2_-C=O), 33.87 (1C, NH-CH_2_-CH_2_-**C**H_2_-C=O), 38.55 (1C, NH-**C**H_2_-CH_2_-CH_2-_C=O), 51.60 (1C, O**C**H_3_), 119.41 (1C, phenyl **C2**), 119.44 (1C, phenyl **C5**), 120.45 (1C, CH=**C**H-C=O), 125.77 (1C, phenyl **C6**), 131.72 (1C, phenyl **C1**), 147.78 (1C, **C**H=CH-C=O), 150.02 (1C, phenyl **C3**), 152.20 (1C, phenyl **C4**), 169.94 (1C, N-**C**=O), 177.57 (1C, **C**OOCH_3_). Anal. Calcd for C_14_H_17_ΝO_5_: C, 60.21; H, 6.14; Ν, 5.02. Found: C, 60.42; H, 5.87; Ν, 5.14.

### 3.2. Biological Experiments

#### 3.2.1. In Vitro Lipid Peroxidation

The peroxidation of heat-inactivated rat liver microsomal fraction was induced by ferrous ascorbate. The studied compounds dissolved in dimethylsulfoxide were added at concentrations between 1 μM–1 mM. Aliquots were taken from the incubation mixture (37 °C) for 45 min. Lipid peroxidation was assessed spectrophotometrically (535/600 nm) as per the method using 2-thiobarbituric acid as the reagent. All compounds and solvents were found not to interfere with the assay [32].

#### 3.2.2. In Vitro Interaction with Stable Radical 1,1-diphenyl-2-picrylhydrazyl (DPPH)

Compounds (dissolved in absolute ethanol, final concentrations 50–200 μM) were added to an ethanolic solution of DPPH (final concentration 200 μM) at room temperature (22 ± 2 °C). Absorbance at 517 nm was recorded after 30 min against a blank sample [32].

#### 3.2.3. Carrageenan-Induced Paw Edema

A total of 0.1 mL of an aqueous carrageenan solution (1% *w*/*v*) was injected into the hind paw of rats. The tested compounds (in water with a few drops of Tween 80) were given i.p. (0.15 mmol/kg), 5 min before the carrageenan administration. The produced edema was measured after 3.5 h, as paw weight increase [32].

#### 3.2.4. In Vitro Evaluation of Lipoxygenase Activity

The reaction mixture contained the test compounds (in absolute ethanol, 100 μM), soybean lipoxygenase (in saline, 250 U/mL) and sodium linoleate (100 μM), in Tris–HCl buffer, pH 9.0. The reaction was followed for 7 min at 28 °C, recording the absorbance at 234 nm. Nordihydroguaiaretic acid was used as a reference. For the estimation of the type of inhibition, the described experiments were repeated, using sodium linoleate at a concentration (1 mM) higher than the saturating substrate concentration [53].

#### 3.2.5. Effect on Plasma Cholesterol and Triglyceride Levels

Hyperlipidemia was induced by the i.p. administration of Triton WR 1339 (200 mg/kg) to rats. The examined compounds (0.15 mmol/kg) were administered i.p. one hour later. Blood was taken from the aorta after 24 h for the determination of total cholesterol and triglyceride concentration using commercial kits [53].

### 3.3. In Silico Calculations

The computational methods used for the theoretical description of the antioxidant potential of polyphenols lie in the possibility to predict their molecular behavior. In this context, we have undertaken an investigation focusing on the influence of structures of the present compounds. For all compounds, the theoretical quantum parameters, specifically the Highest Occupied Molecular Orbital (HOMO) and Lowest Unoccupied Molecular Orbital (LUMO), were computed. Meanwhile, for compounds **1**, **2** and **6**, supplementary computation of the Bond Dissociation Enthalpy (BDE) calculations was carried out for phenol groups. This aimed to bridge and explain the results of the in vitro antioxidant assays when related to their structure.

The HOMO–LUMO gap provides insights into the electron-donating and -accepting capabilities of antioxidants, shedding light on their redox properties. Additionally, the BDE analysis specifically targets the strength of bonds associated with hydroxyl groups—a crucial determinant of the compounds’ ability to donate hydrogen atoms and quench free radicals.

The literature indicates that the hydrogen atom transfer (HAT) mechanism, involving the homolytic break of the O-H bond and the simultaneous release of a hydrogen atom (comprising a proton and an electron), is a pivotal pathway when polyphenols act as antioxidants. Because of that, this mechanism has been thoroughly examined in our current research. Schematization of the HAT mechanism would be the chemical reaction Ar-OH → Ar-O• + H•. Dependent on the structure of the compounds, the ease of O-H bond breaking and consequent release of H• are strongly linked to their antiradical properties and to the ease of conjugating the resulting odd electron from the phenoxy radical over the molecule to gain a low-energy state. Contrarily, mechanisms like single electron transfer–proton transfer (SET–PT) and sequential proton loss electron transfer (SPLET) are considered less favorable for phenolic compounds and were not evaluated in the present study [55,56,57]. The computations were conducted using Spartan20 (Wavefunction, CA, USA) on the B3LYP level of theory with the 6-311++G(d,p) basis set for compounds **1**–**6** in vacuum, a non-polar solvent and water, to obtain information regarding how the polarity of the solvent would influence the antioxidant activity of the compounds [55,58,59].

## 4. Conclusions

The rationale for the design and synthesis of the described compounds was to yield structures with at least two or more potent pharmacological characteristics (antioxidant, anti-inflammatory and hypolipidemic). The aim was to study pleiotropically acting derivatives that would be able to affect, in various aspects, the progression of multicausal conditions implicating the immune, cardiovascular and nervous systems. 

Compounds **1**–**6** were shown to possess at least two of these characteristics. Dual antioxidant moieties in one structure (compound **1**) showed improved an antioxidant effect and good anti-inflammatory and hypolipidemic potency, whilst a pluripotent effect was also recorded in the case of compound **6**, a caffeic acid and GABA hybrid. The results of compound **2** confirm its pluripotency that succeeded in possessing antioxidant, anti-inflammatory and hypolipidemic characteristics, with the incorporation of one NSAID and one antioxidant in one structure, whilst the insertion of proline and cinnamic acid seemed to offer improvement in the cholesterol and triglyceride levels and inflammation reduction.

The analysis of frontier molecular orbitals provided some preliminary information regarding the electron properties of the compounds, but they failed in describing the antioxidant activity of the compounds related to their structure. Thus, more complex calculations such as the bond dissociation enthalpy were performed for phenolic antioxidants, because they capture the full complexity of antioxidant mechanisms and other factors such as molecular structure and functional groups with antioxidant properties.

Taking into account that multi-drug therapies may account for increased adverse effects and pharmacokinetic and pharmacodynamic interactions, the development of structures combining several actions in one molecule may assist towards efficient and safe drugs.

## Figures and Tables

**Table 1:** Effect of the synthesized compounds **1–6** and Trolox on rat microsomal membrane lipid peroxidation.

Compound	Inhibition of Lipid Peroxidation: IC_50_ (μΜ) ^#^
**1**	88
**2**	235
**3**	-
**4**	-
**5**	-
**6**	92
**Trolox**	25

^#^ After 45 min of incubation; Trolox: 6-hydroxy-2,5,7,8-tetramethylchroman-2-carboxylic acid; -: practically inactive or of very low potency. All determinations were performed in triplicate and the standard deviation was always within ±10% of the mean value.

**Table 2:** Calculated physicochemical properties of the synthesized compounds: molecular weight MW), total polar surface area (TPSA), number of H donors and acceptors, lipophilicity (clog*P*) and violations of “Lipinski’s rule of 5”.

Compound	MW	TPSA (Å^2^)	clog*P*	H Acceptors	H Donors	Violations
**1**	427.53	95.86	5.05	6	3	1
**2**	438.90	87.66	5.46	6	3	1
**3**	372.85	58.64	4.81	5	1	0
**4**	267.32	38.66	3.44	3	0	0
**5**	327.37	57.12	3.10	5	0	0
**6**	279.29	95.86	1.17	6	3	0

TPSA and clog*P* calculated with ChemBioDraw Ultra 12.0.

**Table 3:** Interaction of compounds **1**–**6** and Trolox, at various concentrations, with DPPH (200 μΜ) ^a^.

Compound	Percentage Interaction with DPPH			
	200 μΜ	100 μΜ	50 μΜ	25 μΜ
**1**	52.59	39.61	33.22	11.15
**2**	31.36	17.56	7.97	5.40
**3**	-	-	-	-
**4**	6.01	-	-	-
**5**	5.02	-	-	-
**6**	91.80	90.10	47.40	22.00
**Trolox**	92.00	90.00	38.00	10.50

^a^ After 30 min of incubation. Trolox: 6-hydroxy-2,5,7,8-tetramethylchroman-2-carboxylic acid; -: inactive. All determinations were performed in triplicate and standard deviation is always within ±10% of the mean value.

**Table 4:** Effect of compounds **1**–**6**, ibuprofen and tolfenamic acid on carrageenan-induced rat paw edema ^a^.

Compound	% Edema Reduction
**1**	49 ***
**2**	59 ***
**3**	35 **
**4**	38 ***
**5**	25 *
**6**	48 ***
**Ibuprofen**	36 **
**Tolfenamic acid**	24 ***

^a^ The effect on edema is expressed as percentage inhibition of edema in comparison to controls. All compounds were administered i.p. at a dose of 0.15 mmol/kg of body weight. Each value represents the mean obtained from five to six animals. Significant difference from control: * *p* < 0.05, ** *p* < 0.005, *** *p* < 0.001 (Student’s *t*-test).

**Table 5:** Inhibitory activity of compounds **1**–**6**, tolfenamic acid and nordihydroguaiaretic acid (concentration 100 μM) against soybean lipoxygenase ^a^.

Compound	% Inhibition
**1**	28
**2**	56
**3**	-
**4**	20
**5**	34
**6**	10
**Tolfenamic acid**	15
**NDGA**	94

^a^ After 7 min of incubation; NDGA: nordihydroguaiaretic acid; -: inactive. All determinations were performed in triplicate and the standard deviation was always within ±10% of the mean value.

**Table 6:** Effect of the synthesized compounds **1**–**6**, together with simvastatin and tolfenamic acid (dose 150 μmol/kg i.p.), on Triton WR1339 (tyloxapol)-induced hyperlipidemia.

Compound	% Reduction	
	TC ^a^	TG ^b^
**1**	38.5 **	42.3 *
**2**	75.1 **	54.5 **
**3**	67.4 ***	79.3 ***
**4**	53.0 ***	47.0 **
**5**	58.1 ***	59.6 ***
**6**	55.1 ***	44.03 ***
**Simvastatin**	73.0 ***	-
**Tolfenamic acid**	19.0 *	17.0 *

^a^ TC: total cholesterol; ^b^ TG: triglycerides. Tyloxapol: 200 mg/kg, i.p.: not statistically significant result. Significant difference from hyperlipidemic control group: * *p* < 0.03, ** *p* < 0.005, *** *p* < 0.001 (Student’s *t*-test).

**Table 7:** Frontier molecular orbitals HOMO, LUMO and the HOMO–LUMO gap in vacuum, non-polar environment and water (eV).

Compound	Vacuum			Non-Polar			Water		
	HOMO	LUMO	Gap	HOMO	LUMO	Gap	HOMO	LUMO	Gap
**1**	−5.76	−0.60	5.16	−5.77	−0.59	5.18	−5.89	−0.86	5.03
**2**	−5.35	−0.66	4.69	−5.30	−0.52	4.78	−5.39	−0.64	4.75
**3**	−5.39	−1.05	4.34	−5.35	−0.96	4.39	−5.45	−1.09	4.36
**4**	−6.40	−1.76	4.64	−6.33	−1.72	4.61	−6.39	−1.88	4.51
**5**	−5.79	−1.56	4.23	−5.72	−1.55	4.17	−5.88	−1.80	4.08
**6**	−5.76	−1.47	4.29	−5.63	−1.34	4.29	−5.65	−1.49	4.16

**Table 8:** The graphical depiction of frontier molecular orbitals HOMO, LUMO in vacuum.

1	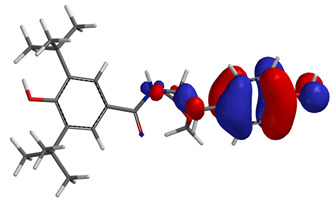	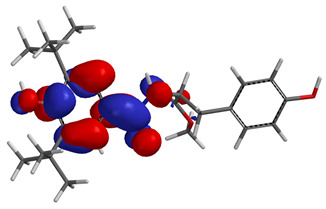
2	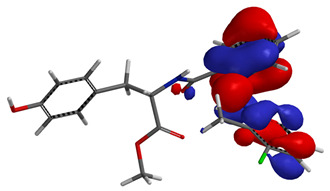	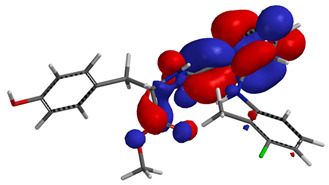
3	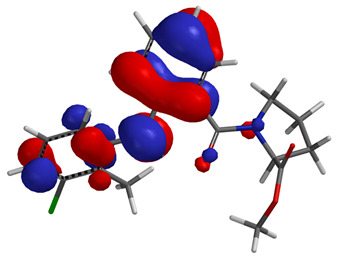	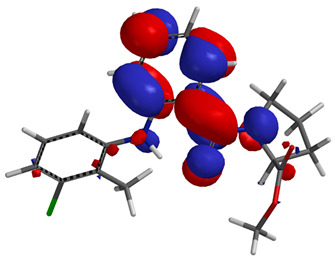
4	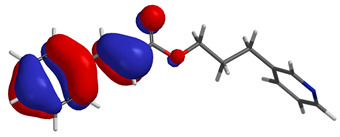	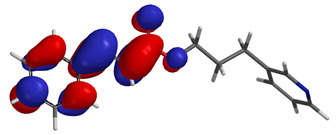
5	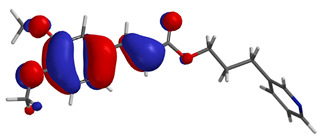	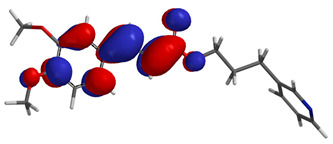
6	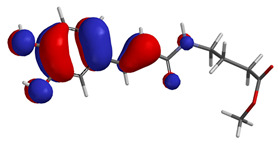	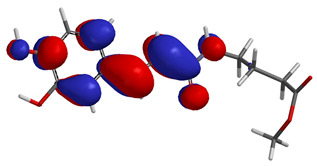

**Table 9:** BDE for the OH groups from compounds **1**, **2** and **6** in vacuum, non-polar environment and water (kcal/mol).

Compound	OH Group Localization	Vacuum	Non-Polar	Water
**1**	tyrosine	77.97	77.02	77.80
	di-*tert*-butylphenol	72.58	72.22	74.41
**2**	tyrosine	78.23	77.55	77.97
**6**	*para*	67.51	67.69	70.25
	*meta*	70.12	70.49	73.48

## Data Availability

Data are contained within the article.
